# Endoscopic suturing with muscular after fundus mucoresection for refractory gastroesophageal reflux disease：a new anti-reflux technique

**DOI:** 10.1055/a-2663-8344

**Published:** 2025-08-20

**Authors:** Shan Wu, Meiying Zhu, Xinjian Wan

**Affiliations:** 1 378725 Digestive Endoscopic Center, Shanghai Sixth Peopleʼs Hospital Affiliated to Shanghai Jiaotong University, Shanghai, China

A 63-year-old male with a 20-year history of proton pump inhibitor (PPI)-refractory gastroesophageal reflux disease (GERD) presented with persistent heartburn and regurgitation (GERD-HRQL score 29). High-resolution manometry showed a hypotensive lower esophageal sphincter (LES), and 24-hour pH-impedance monitoring confirmed pathological reflux (DeMeester score: 83.18). The patient underwent endoscopic suturing with muscular after fundus mucoresection (ESFM), a novel hybrid procedure combining endoscopic mucosal resection with fundoplication principles ( Fig. 1 ). Following an 8-hour preoperative fasting period, the gastric cardia was endoscopically exposed. Key steps included are as follows: mucosal marking at the gastric cardia (1.5 cm × 2 cm area); submucosal injection and circumferential endoscopic mucosal resection; nylon loop placement with clip-anchored muscular plication; and creation of an anti-reflux valve via suture tightening ( Video 1 ). Postoperative recovery was uneventful with a 3-day hospitalization. At 3-month follow-up, the patient reported complete resolution of reflux symptoms without dysphagia (GERD-HRQL 7). Repeat endoscopy demonstrated a well-healed plication fold.

**Fig. 1:**
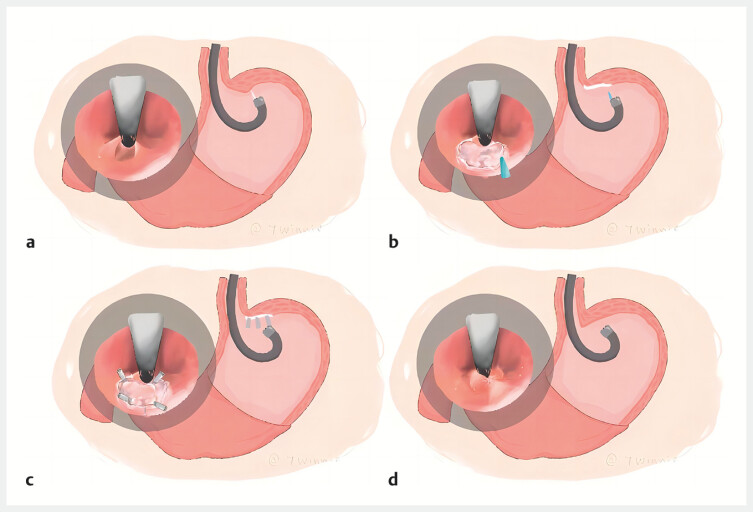
Endoscopic suturing with muscular after fundus mucoresection (ESFM) procedure for PPI-refractory GERD. **a** Preoperative view showing a patulous cardia. **b** Mucosal resection at the gastric fundus. **c** Suturing with muscular layer plication. **d** Postoperative appearance demonstrating successful luminal narrowing.

Endoscopic suturing with muscular after fundus mucoresection (ESFM).Video 1

The ESFM procedure effectively addresses GERD pathophysiology by creating a mechanical barrier through combined mucosal resection and muscular plication while narrowing the lumen at the esophagogastric junction, avoiding the morbidity associated with traditional surgical interventions 1 2 . This case demonstrates ESFM as a promising minimally invasive alternative for PPI-refractory GERD, successfully merging the safety profile of endoscopic resection with the functional efficacy of fundoplication 3 . Further studies are necessary to validate the long-term clinical outcomes of this innovative technique.

Endoscopy_UCTN_Code_TTT_1AO_2AJ

## Notes

Endoscopy E-Videos https://eref.thieme.de/e-videos
*E-Videos* is an open access online section of the journal *Endoscopy* , reporting on interesting cases and new techniques in gastroenterological endoscopy. All papers include a high-quality video and are published with a Creative Commons CC-BY license. Endoscopy E-Videos qualify for HINARI discounts and waivers and eligibility is automatically checked during the submission process. We grant 100% waivers to articles whose corresponding authors are based in Group A countries and 50% waivers to those who are based in Group B countries as classified by Research4Life (see: https://www.research4life.org/access/eligibility/ ). This section has its own submission website at https://mc.manuscriptcentral.com/e-videos .
